# RNA-Seq analysis of salinity stress–responsive transcriptome in the liver of spotted sea bass (*Lateolabrax maculatus*)

**DOI:** 10.1371/journal.pone.0173238

**Published:** 2017-03-02

**Authors:** Xiaoyan Zhang, Haishen Wen, Hailiang Wang, Yuanyuan Ren, Ji Zhao, Yun Li

**Affiliations:** The Key Laboratory of Mariculture (Ocean University of China), Ministry of Education, Ocean University of China, Qingdao, P. R. China; Xiamen University, CHINA

## Abstract

Salinity is one of the most prominent abiotic factors, which greatly influence reproduction, development, growth, physiological and metabolic activities of fishes. Spotted sea bass (*Lateolabrax maculatus*), as a euryhaline marine teleost, has extraordinary ability to deal with a wide range of salinity changes. However, this species is devoid of genomic resources, and no study has been conducted at the transcriptomic level to determine genes responsible for salinity regulation, which impedes the understanding of the fundamental mechanism conferring tolerance to salinity fluctuations. Liver, as the major metabolic organ, is the key source supplying energy for iono- and osmoregulation in fish, however, little attention has been paid to its salinity-related functions but which should not be ignored. In this study, we perform RNA-Seq analysis to identify genes involved in salinity adaptation and osmoregulation in liver of spotted sea bass, generating from the fishes exposed to low and high salinity water (5 vs 30ppt). After *de novo* assembly, annotation and differential gene expression analysis, a total of 455 genes were differentially expressed, including 184 up-regulated and 271 down-regulated transcripts in low salinity-acclimated fish group compared with that in high salinity-acclimated group. A number of genes with a potential role in salinity adaptation for spotted sea bass were classified into five functional categories based on the gene ontology (GO) and enrichment analysis, which include genes involved in metabolites and ion transporters, energy metabolism, signal transduction, immune response and structure reorganization. The candidate genes identified in *L*. *maculates* liver provide valuable information to explore new pathways related to fish salinity and osmotic regulation. Besides, the transcriptomic sequencing data supplies significant resources for identification of novel genes and further studying biological questions in spotted sea bass.

## Introduction

Salinity is one of the most important environmental factors that greatly affect the survival, reproduction, growth, development and physiological functions of teleosts. The tolerance to salinity variation depends on their ability of osmotic regulation [1]. Euryhaline teleosts, which are usually exposed to constant and rapid changes in the salinity of external environment, have the superior ability for osmoregulation and can tolerate a wide range of external salt concentrations [2]. The spotted sea bass, *Lateolabrax maculatus*, belonging to *Lateolabrax*, Serranidae, is a newly re-described euryhaline teleost specie which usually inhabits in estuary brackish-water, and can also live in fresh water [3,4]. Previous studies have revealed that *L*. *maculatus* grows in optimal salinity ranged from 16–17 ppt, whereas they can survive at salinities below 38 ppt and above 0 ppt [5]. To cope with water osmotic pressures caused by the change of tide, terrigenous drainage and climate, *L*. *maculates*, like other euryhaline fishes, has evolved unique and complex osmotic regulation mechanism [6]. Euryhaline species also enable comparative studies in which one environment can be used as a standard to contrast the specific genes or pathways for freshwater or saltwater osmoregulation [7]. Therefore, together with its economical importance, spotted sea bass is a valuable template for investigating the mechanisms of acclimation to osmotic stress and the responses to salinity changes. Moreover, as the coastal cage culture of *L*. *maculates* suffers from germplasm degeneration, many inland areas began to explore freshwater domestication of *L*. *maculates* [8]. Thus understanding the mechanism underlying the adaptation to fluctuated environmental salinity can support the regional culture industry and further expansion of the culture of *L*. *maculatus*.

Various enzymes and transporters participate in the processes of salinity adaption and osmoregulation to maintain an internal osmotic and ionic homeostasis when live in a salinity fluctuating environment [9,10]. Identification of candidate genes involved in salinity change is the first step to elucidate the molecular basis and understand factors underlying this core physiological process. As the transcriptome is a dynamic set of expressed genes that is sensitive to the external environment and will change depending on the physiological conditions, transcriptomic analysis is a powerful tool for interpreting the functional genomics elements and for revealing molecular mechanisms in cells and tissues [11]. Next generation high-throughput RNA sequencing technology (RNA-Seq) now provides a cost-and time-effective way to generate transcriptomic resources to find and determine the putative genes and gene families responsible for stress response in species [12–14]. RNA-Seq can also provide reliable measurements of transcripts in one or more conditions, which make it particularly attractive for the quantitative analysis of gene expression in transcriptomic level [15]. Over the last several years, significant progress has been made in understanding the transcript expression profile during salinity adaptation of various euryhaline teleost species by RNA-Seq, such as nile tilapia (*Oreochromis niloticus*), Mozambique tilapia (*Oreochromis mossambicus*) [7], medaka (*Oryzias melastigma)* [16], striped catfish (*Pangasianodon hypophthalmus*) [11,17] and Asian seabass (*Latescal carifer*) [18]. Those studies highlighted several differentially expressed genes (DEGs) and pathways involved in salinity change. However, such investigations in *L*. *maculatus* have not been reported. Previous studies just examined the growth, development, reproductive activities and physiology behaviors exposed to different salinities [5,8], the research on the molecular mechanism of acclimation to salinity and osmotic stress in *L*. *maculatus*, actually, legs far behind.

A timely and adequate energy supply is a prerequisite for iono- and osmoregulation in fish, while carbohydrate metabolism plays a major role in the energy supply [19]. Liver, as the most fundamental metabolic organ, has proved to be the major source supplying carbohydrate metabolites to osmoregulatory organs [20]. Studies further suggested that the liver could promote the decomposition of glycogen into glucose in order to maintain normal blood sugar levels of fish, as well as provide the energy required by gill and other osmoregulatory organs under the salinity stress [21, 22]. However, despite its importance, the role of liver in fish osmoregulation is hardly known compared with osmoregulatory organs (such as gill, kidney and intestine) which have received considerable attention over the years [7, 23]. Thus, for osmoregulation study in fish, a new window should be opened for examining the activities occurring in liver and explore its role in salinity adaption.

As salinity stress in spotted sea bass remains largely unexplored and the genomic and transcriptomic data available for this species is still scarce, in this study, we conducted RNA-Seq to identify and characterize salinity-induced genes expressions in liver tissue of *L*. *maculatus*. Our results will provide a valuable resource for a better understanding of osmoregulatory process of spotted sea bass. The candidate genes identified in this study would serve as potential list for gene-assisted genetic breeding program for spotted sea bass as well as other euryhaline species.

## Materials and methods

### Ethics statement

All animal experiments were conducted in accordance with the guidelines and approval of the respective Animal Research and Ethics Committees of Ocean University of China. The field studies did not involve endangered or protected species.

### Salinity challenge and fish sampling

Spotted sea bass fingerlings (10.66 ± 0.05 g) were acquired from Doumen district river fishery research institute (Zhuhai city, Guangdong province, China). Fish were maintained under a 14:10 h light–dark photoperiod in a 3m*4m*0.5m cement tank for one week before experiment. Water temperature, dissolved oxygen, salinity and pH were maintained at 25.3±0.7°C, 7.01 ±0.45mg/ L, 20±0.8‰ and 7.8±0.5, respectively. After acclimation, fingerlings were randomly assigned to two groups: low salinity group (LS, 5ppt) and high salinity group (HS, 30ppt) in 6 tanks, all treatment groups were triplicated. After 60 days breeding, 6 fishes per tank were treated with tricaine methane sulfonate (MS 222, 200 mg/L) and sampled immediately. Liver tissues were collected and stored at -80°C for RNA extraction.

### RNA extraction, library construction and transcriptome sequencing

Total RNA was isolated from liver samples using the TRIzol^®^ reagent (Invitrogen, USA) and treated with the TURBO DNA- free^™^ kit (Invitrogen) to remove genomic DNA. The concentration and integrity of total RNA were assessed using the Agilent 2100 Bioanalyzer system (Agilent Technologies, USA). In order to minimize the variation among individuals, equal amounts of RNA from 6 individual fish in the same salinity tank were pooled as one sample, and 3 replicated samples were made for each salinity treatment group. A total of 6 sequencing libraries (3 replicated samples X 2 treatment groups) were generated using NEBNext^®^ Ultra^™^ RNA Library Prep Kit for Illumina^®^ (NEB, USA) following manufacturer’s recommendations and index codes were added to attribute sequences to each sample. Samples were then sequenced on an IlluminaHiseq 2500 platform and 125 bp paired-end reads were generated. Raw sequences were deposited in the Short Read Archive of the National Center for Biotechnology Information (NCBI) with accession numbers of SRR4409341 (LS) and SRR4409397 (HS).

### *De novo* assembly of sequencing reads

Raw data of FASTQ format were firstly processed through in-house perl scripts. In this step, clean data were obtained by removing reads containing adapter, reads containing ploy-N and low quality reads from raw data. Q20, Q30, GC-content and sequence duplication level of the clean data were calculated. All the downstream analyses were based on clean data with high quality.

The *de novo* assembly was performed on liver clean reads using Trinity package [24]. Briefly, the reads were assembled into unique sequences of transcripts in *Inchworm* via greedy K-mer extension (K-mer = 25). After mapping of reads to *Inchworm* contigs, *Chrysalis* incorporated reads into *de bruijn* graphs. Finally the *Butterfly* module processed the individual graphs in parallel, generating full-length transcripts. This Transcriptome Shotgun Assembly project has been deposited at DDBJ/EMBL/GenBank under the accession GFDU00000000. The version described in this paper is the first version, GFDU01000000.

### Annotations of transcripts and pathways

The assembled transcripts were scanned against the Nr (NCBI non-redundant protein sequences), Nt (NCBI nucleotide sequences) and Swiss-Prot databases using BLASTX [25] with E-values at 1.0×10^−5^ (E-values less than 1.0×10^−5^ were considered as significant). Domain-based comparisons with Pfam (Protein family) and KOG (a eukaryote-specific version of the Clusters of eukaryotic Ortholog Groups) databases were performed using RPS-BLAST tool from locally installed NCBI BLAST + v2.2.28 and HMMER 3.0 program, respectively. The top gene identifications and names were initially assigned to each transcript. In addition, transcripts were used to determine the Gene Ontology (GO) term and the Kyoto Encyclopedia of Genes and Genomes (KEGG) pathway. GO enrichment (Biological Process, Cellular Component and Molecular Function at level 2) was performed using Blast 2GO program [26]. The overview of metabolic pathway analysis was performed using online KEGG (http://www.genome.jp/kegg/), which is a database [27] resource for understanding high-level functions and utilities of the biological system.

### Gene differential expression and enrichment analysis

The high-quality clean reads from each library were mapped to the *de novo* assembled transcripts using bowtie 2 program [28] with no mismatch. The mapped reads from alignments were counted and then normalized to determine FPKM (expected number of Fragment Per Kilobase of transcript sequence per Millions base pairs sequenced) using RSEM V1.2.15 [29]. Differential expression statistical analysis of two experimental groups (LS and HS) was performed using the DESeq R package [30]. An adjusted p-value cut off of 0.05 was applied in this analysis. Transcripts with absolute fold change values higher than 2.0 were regarded as significantly differential expressed genes. Gene Ontology (GO) enrichment analysis of the differentially expressed genes (DEGs) was implemented by the GOseq packages based on Wallenius non-central hyper-geometric distribution [31], which can adjust for gene length bias in DEGs.

### Experimental validation by qRT-PCR

Quantitative real-time PCR (qRT-PCR) was used to detect the expression of 10 differential expressed genes to validate our Illumina sequencing data. RNA samples were generated from HS and LS groups (with three replicate samples each group) in the preceding experiment. Primers were designed based on the assembled transcriptome sequence using the Primer5 software (Premier Biosoft International) and listed in S1 Appendix. The first strand cDNA was synthesized from 1 μg of RNA by using M-MLV Reverse Transcription Kit (Promega, USA). All the cDNA products were diluted to 200ng/μl. The 20 μl qRT-PCR reaction mixture consisted of 2μl template cDNA, 0.4μl of each primer, 10 μl of KAPA SYBR^®^FAST qPCR Master Mix (2X), 0.4μl of ROX and 6.8μl of nuclease-free water. PCR amplification was performed as that incubated in a 96-well optical plate at 95°C for 2 min, followed by 40 cycles of 95°C for 15s, 56°C for 15s, and a final extension at 72°C for 2 min. qRT-PCR was performed using the StepOne Plus Real-Time PCR system (Applied Biosystems) and 2^-ΔΔCT^ method was used to analysis the expression level of genes. *18S* ribosomal RNA (*18S*) was used as the reference gene for qRT-PCR normalization. The correlation coefficient between the fold changes in RNA-Seq group and qRT-PCR group was determined by SPSS13.0, one-way ANOVA followed by Duncan’s multiple range tests and differences were accepted as statistically significance when P < 0.05.

## Results

### *De novo* assembly for spotted sea bass

RNA-Seq was carried out on liver samples from two groups with different salinity concentrations (LS /5ppt and HS /30ppt). A total of 367,900,812 raw reads (125 bp) were obtained from the six liver samples on the Illumina HiSeq 2500 platform. After preprocessing and removal of low-quality sequences, a total of 351,647,998 clean reads were generated (Table 1), with 180 million qualified clean reads for the LS samples and 172 million for HS samples (Table 2).

**Table 1:** Summary of statistics for Illumina short reads of the liver transcriptome of spotted sea bass.

Samples^a^	Raw reads	Clean reads	Q20(%)^b^	Q30(%)^c^	Total Mapped (%)^d^
**LS_1**	65,774,378	63,254,776	94.98	90.63	53,572,246(84.69%)
**LS_2**	61,987,790	59,733,204	94.78	90.39	50,123,822(83.91%)
**LS_3**	60,765,056	56,932,004	93.93	89.26	44,928,850(78.92%)
**HS_1**	56,833,448	53,399,110	94.77	90.45	43,566,350(81.59%)
**HS_2**	66,351,972	64,318,628	95.34	91.14	54,720,808(85.08%)
**HS_3**	56,188,168	54,010,276	94.65	90.21	43,844,928(81.18%)
**Total**	367,900,812	351,647,998			

^**a**^1, 2 and 3: Three independent biological replicates;

^**b**^Q20: The percentage of bases with a Phred value > 20;

^**c**^Q30: The percentage of bases with a Phred value > 30;

^**d**^The number of clean reads that mapped onto the assembled reference transcriptome.

**Table 2:** Summary of assembly and annotation statistics of the liver transcriptome of spotted sea bass.

Category	Number of transcripts
Total number of clean reads of LS	179,919,984
Total number of clean reads of HS	171,728,014
Average length of all transcripts (bp)	747
N50 length of all transcripts (bp)	1,536
Max length (bp)	20,355
Min length (bp)	201
Total number of annotated transcripts in Nr database	33,877 (17.14%)
Total number of annotated transcripts in Nt database	45,293 (22.92%)
Total number of annotated transcripts in Pfam database	30,854 (15.61%)
Total number of annotated transcripts in KEGG database	17,046 (8.62%)
Total number of annotated transcripts in KOG database	14,905 (7.54%)
Total number of annotated transcripts in GO database	31,073 (15.72%)
Total number of annotated transcripts in Swiss-Prot database	26,511 (13.41%)
Total number of annotated transcripts in at least one database	60,644 (30.69%)

After *de novo* assembly analysis based on all Illumina clean reads, a total of 197,550 transcripts (ranging from 201 to 20,355bp) were generated for *L*. *maculates* with N50 size of 1,536 bp (Table 2). For all six sequencing libraries, the percentages of reads that could be mapped to assembled reference sequences were higher than 78.90% (Table 1).

### Annotation and function analysis of liver transcripts

Transcripts were subjected to annotation analysis by comparing with Nr, Nt, Pfam, KOG, Swiss-Prot, KEGG and GO databases. Results show that a total of 60,644 transcripts (30.69%) were annotated in at least one database, with 33,877 annotated transcripts (17.15%) had a significant BLAST hit against Nr database. The detailed annotation results are listed in Table 2.

For top-hit species matched against Nr database, 42.6% of the matched transcprits showed similarities with *Stegastes partitus*, followed by *Oreochromis niloticus* (9.6%), *Haplochromis burtoni* (4.5%), *Neolamprologus brichardi* (4.5%), *Cynoglossus semilaevis* (3.8%), and others (35%) (S2A Appendix).

The GO analysis of the above annotated transcripts demonstrated that a total of 31,073 (15.73%) liver transcripts of spotted sea bass were assigned to 49 GO terms including 13 molecular function (MF) terms, 15 cellular component (CC) terms and 21 biological processes (BP) terms. Analysis of level 2 GO term distribution showed that cellular process (GO: 0009987), metabolic process (GO: 0008152), single-organism process (GO: 0044699), biological regulation (GO: 0065007) and regulation of biological process (GO: 0050789) in BP, cell (GO: 0005623), cell part (GO: 0044464), organelle (GO: 0043226), macromolecular complex (GO: 0032991), membrane (GO: 0016020), membrane part (GO: 0044425) and organelle part (GO: 0044422) in CC, and binding (GO: 0005488), catalytic activity (GO: 0003824) and transporter activity (GO: 0005215) in MF were the most common annotation terms in the three GO categories (S2B Appendix).

In order to assess and classify possible functions, assembled transcripts were aligned to the KOG database in which orthologous gene products were classified. A total of 14,905 (7.54%) transcripts were assigned to 26 KOG categories. Among the matched sequences, 3,378 transcripts were assigned into the KOG category of signal transduction mechanisms, which represented the largest functional group, followed by general function prediction (2,850), posttranslational modification, protein turnover, chaperones (1,405), transcription (1,080), intracellular trafficking, secretion, and vesicular transport (961), cytoskeleton (890) and other classifications with smaller numbers of transcripts (S2C Appendix).

KEGG pathway analysis was performed to further elucidate the probable functional status of assembled transcripts. A total of 17,046 (8.63%) annotated liver transcripts of spotted sea bass with significant matches were assigned to five main categories that included 271 KEGG pathways. Among the five main categories that were identified, organismal systems held the greatest number of transcripts (6,678), followed by environmental information processing (4,262), metabolism (3,406), cellular processes (3,153) and genetic information processing (1,838). As shown in S2D Appendix, the five main categories contained 32 sub-categories, transcripts of which were significantly engaged in signal transduction (3,125), endocrine system (1,366), immune response (1,279), cellular community (1,129) and signal molecule interactions (1,019).

### Identification of differentially expressed genes

In the liver of spotted sea bass, a total of 455 annotated transcripts showed significantly differential expression between the two salinity challenged groups (LS vs HS) (adjusted p-value < 0.05), of which 184 genes were up-regulated and 271 genes showed down-regulated in the LS group relative to HS group (S3 and S4 Appendices).

GO enrichment analysis of the 455 DEGs was performed from three aspects including CC, MF and BP. Among these categories, most DEGs were enriched in the "cellular component" category (S5 Appendix), which include 92 transcripts in membrane (GO: 0016020) term, 59 transcripts in membrane part (GO: 0044425) term, 48 transcripts in integral to membrane (GO: 0016021) term and 48 transcripts in intrinsic to membrane (GO: 0031224) term. Regarding the “BP” and "MF" category, only a few transcripts were enriched. For example, the three most abundant subcategories in “BP” category including cell adhesion term (GO: 0007155, 15 transcripts), biological adhesion term (GO: 0022610, 15 transcripts), and macromolecular complex assembly term (GO: 0065003, 10 transcripts). As for the "MF" category, most DEGs were assigned to 23 peptidase activity that acting on L-amino acid peptides term (GO: 0070011, 23 transcripts), peptidase activity term (GO: 0008233, 23 transcripts), endopeptidase activity term (GO: 0004175, 18 transcripts) and receptor activity term (GO: 0004872, 17 transcripts) (S5 Appendix).

KEGG pathway analysis showed that the DEGs were engaged in several specific pathways, such as renin-angiotensin system, glycosaminoglycan biosynthesis-keratan sulfate, hepatitis C, vitamin digestion and absorption, mucin type O-Glycan biosynthesis and toll-like receptor signaling pathway. The top 15 most enriched KEGG pathways are shown in Table 3

**Table 3:** The top 15 enriched KEGG pathways in the DEGs.

Pathway terms	KEGG ID	DEGs number	Background number^a^	P-Value
Renin-angiotensin system	ko04614	4	23	8.13E-05
Glycosaminoglycan biosynthesis—keratan sulfate	ko00533	4	28	0.000161
Hepatitis C	ko05160	7	220	0.003718
Vitamin digestion and absorption	ko04977	3	41	0.006466
Mucin type O-Glycan biosynthesis	ko00512	3	42	0.006884
Hypertrophic cardiomyopathy (HCM)	ko05410	6	190	0.007295
Legionellosis	ko05134	4	106	0.015311
Glutathione metabolism	ko00480	3	60	0.017203
Glycosphingolipid biosynthesis—globo series	ko00603	2	22	0.018024
Hematopoietic cell lineage	ko04640	3	65	0.021042
Glycosphingolipid biosynthesis—ganglio series	ko00604	2	25	0.022538
Amino sugar and nucleotide sugar metabolism	ko00520	3	69	0.024423
Tight junction	ko04530	7	323	0.025092
Cysteine and methionine metabolism	ko00270	3	73	0.02808
Leukocyte transendothelial migration	ko04670	6	269	0.03268

^a^Background number: the number of total genes assigned to the pathway

As a non-model species with only limited gene function annotation resources, candidate DEGs potentially associated with salinity adaptation and osmoregulation were categorized in to five functional categories including metabolites and ion transporters, energy metabolism, signal transduction, immune response and structure reorganization (Table 4) based on the combination of enrichment analysis, annotation and manual literature searches. Imputed putative functions of these genes are covered in the **Discussion**.

**Table 4:** Enriched DEGs potentially associated with salinity adaptation and osmoregulation in liver of spotted sea bass.

Functional group	Gene name	Gene ID	log_2_FoldChange (LS vs HS)	Gene function	Cellular component
**Metabolites and Ion transporters**					
	Sodium-coupled monocarboxylate transporter(*slc5a8*,*smct*)	c96993_g1	-6.4	monocarboxylate transport	membrane
	Na/Pi cotransport system protein(*slc34a*, *nptA*)	c34916_g1	-4.9	phosphate ion transport	membrane
	aquaporin 3 (*aqp3*),	c81353_g1	-4.6	Ion transport	membrane
	TRIC channel	c98361_g1	4.811	cation transport	membrane
	solute carrier family 6, monocarboxylate transporters member 15-like (*slc6a15*)	c69992_g1	-4.83	cation transport	integral component of membrane
	ryanodine receptor 2-like	c61290_g1	5.945	calcium ion transport	intracellular
	ATP-sensitive inward rectifier potassium channel(kcnj15)	c96024_g1	-5.07	potassium ion transport	membrane
	transient receptor potential cation channel subfamily M(trpm5)	c99386_g1	-3.73	cation channel	membrane
	apolipoprotein E(Apo-E)	c85127_g1	-2.24	lipid transport	extracellular region
	apolipoprotein B(Apo-B)	c103605_g1	-2.38	lipid transport	extracellular region
	apolipoprotein C(Apo-C)	c186781_g1	-4.75	lipid transport	extracellular region
	transient receptor potential cation channel, subfamily M, member 5 (trpm5)	c99386_g1	-3.73	cation channel	membrane
	Na+-K+-2Cl-contransporter (nkcc2)	c99411_g1	-7.59	transmembrane transport	membrane
	solute carrier family 2, facilitated glucose transporter member 5-like(slc2a5, GLUT5)	c97450_g1	-2.8	glucose transport	integral component of membrane
	solute carrier family 43 member 3-like(slc43a3)	c105513_g1	2.41	transmembrane transport	integral component of membrane
	solute carrier family 39 zinc transporter member 4(slc39a4)	c87485_g1	-2.6708	transmembrane transport	membrane
**Energy metabolism**					
	Glycerophosphodiester phosphodiesterase	c99721_g1	-4.74	lipid metabolic process	-----
	adipose triglyceride lipase(ATGL)	c104357_g1	-2.69	lipid metabolic process	nucleus
	Glycerophosphoinositol inositolphosphodiesterase GDPD	c100119_g1	-3.66	lipid metabolic process	membrane
	Phosphoinosidtide-specific phospholipase(PI-PLC X)	c95974_g1	7.473	lipid metabolic process	membrane
	tyrosine-protein phosphatase non-receptor type substrate 1-like	c186781_g1	-4.75	lipoprotein metabolic process	extracellular region
	Serine proteinase inhibitor	c101702_g1	-4.85	sucrose metabolic process	glucosidase II complex
	betaine-homocysteine S-methyltransferase	c80342_g1	3.053	methionine metabolic process	-----
	acidic mammalian chitinase-like	c1493_g1	-5.37	carbohydrate metabolic process	extracellular region
	Stimulator of interferon genes protein	c99919_g1	3.44	carbohydrate metabolic process	DNA polymerase complex
	G-protein coupled receptor 112-like	c99494_g1	-4.6	lipid metabolic process	membrane
	transmembrane protease serine 9-like	c99970_g1	-3.68	proteolysis	----
	cytochrome P450, family 2	c104946_g2	3.348	oxidation-reduction process	-----
	L-amino-acid oxidase-like	c90095_g1	-3.26	fatty acid metabolic process	cytoplasm
	heme oxygenase-like	c101847_g1	-3.32	oxidation-reduction process	-----
	malate dehydrogenase(MDH)	c94658_g1	-4.06	oxidation-reduction process	-----
	chitinase	c1493_g1	-5.3717	carbohydrate metabolic process	extracellular region
	acyl-CoA synthetase(ACSS, acs)	c92990_g1	-2.869	Tricarboxylic acid cycle	-----
	glucosidase II complex	c49668_g1	3.894	carbohydrate metabolic process	plasma membrane
	Acetyl-CoA acetyltransferase	c99741_g1	-2.6296	lipid biosynthetic process	-----
**Signal transduction**					
	G-protein coupled receptor 64	c82122_g1	-5.655	G-protein coupled receptor signaling	membrane
	Obscurin-like protein 1	c103887_g1	4.0036	glucocorticoid receptor signaling pathway	Nucleus
	cadherin-17-like	c98416_g1	-5.06	signal transduction	extracellular region
	transmembrane protease serine 13-like	c94692_g2	-3.5349	signal transduction	membrane
	ATP P2X receptor	c70881_g1	-4.1795	signal transduction	membrane
	protein phosphatase 1 regulatory subunit 12B-like	c96161_g1	-3.2971	signal transduction	Golgi transport complex
	Kv channel-interacting protein 4-like	c94733_g1	2.9938	signal transduction	proteinaceous extracellular matrix
	ral guanine nucleotide dissociation stimulator-like	c94958_g1	-4.6347	regulation of small GTPase mediated signal transduction	intracellular
	signal transducer and activator of transcription 1	c104292_g1	2.61	signal transduction	nucleus
	uromodulin-like	c103623_g1	-8.43	signal transduction	membrane
	collagen alpha-1	c171794_g1	4.681	signal transduction	intracellular
	peptide Y	c79092_g1	-4.225	signal transduction	extracellular region
	Brain-specific angiogenesis inhibitor 1-associated	c102414_g1	-3.72	signal transduction	-----
	Bone sialoprotein II (BSP-II)	c97890_g2	-3.46	signal transduction	membrane
	Hyaluronan-binding protein 2	c99450_g1	2.386	neuropeptide signaling pathway	protein binding
	protein Wnt-8b	c17833_g1	3.1075	Wnt signaling pathway	extracellular region
	APJ endogenous ligand	c96724_g1	3.015	signal transduction	-----
	nuclear receptor ROR-beta-like	c98760_g1	3.317	signal transduction	nucleus
**Immune response**					
	CD63 antigen	c91869_g1	-5.2891	immune response	integral component of membrane
	complement C1q-like protein 2	c96181_g1	3.4958	immune response	membrane
	interleukin-20 receptor subunit alpha-like	c186794_g1	3.57	immune response	-----
	Colicin E1 (microcin) immunity protein	c82286_g1	-8.5878	immune response	immunoglobulin complex
	Heat-stable enterotoxin receptor	c99469_g1	-3.2616	viral release from host cell	host cell membrane
	interlukin-8	c75114_g1	-2.7554	immune response	extracellular region
	interleukin-4	c100507_g1	-2.54	immune response	extracellular region
**Structure reorganization**					
	claudin-15-like	c11728_g1	-5.5747	structural molecule	plasma membrane
	claudin-4-like	c10230_g1	-4.6235	structural molecule	integral component of membrane
	MAM and LDL-receptor class A domain-containing protein 1	c104114_g1	-4.28	protein binding	membrane
	claudin-8-lik	c103303_g1	-2.7356	structural molecule	integral component of membrane
	PTB domain-containing engulfment adapter protein 1	c35439_g1	-4.26	protein binding	------
	mucin-like protein	c84121_g1	-4.14	protein binding	------
	amphiregulin-like	c88721_g1	-3.53	protein binding	------
	myosin XV	c102433_g1	-3.21	protein binding	cytoskeleton
	myosin VII	c99077_g1	-5.2597	protein binding	cytoskeleton
	fer-1-like protein 4-like	c103495_g2	-5.81	protein binding	integral component of membrane
	alpha-tectorin	c106780_g2	-5.2489	microtubule-based process	microtubule associated complex
	intermediate filament family orphan 1-like	c104224_g2	-2.9968	structural molecule	intermediate filament

### Validation of RNA-Seq results by qRT-PCR

**Fig 1:**
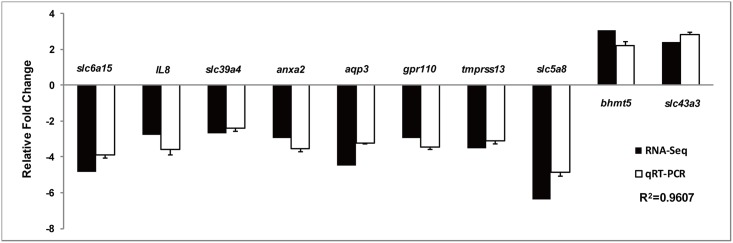
qRT-PCR validation of 10 differentially expressed genes generated from RNA-Seq results in the liver of spotted sea bass. The expression levels of the selected genes were each normalized to that of the *18S* gene. Gene abbreviations are: *slc6a15*, solute carrier family 6 member 15; *slc43a3*, solute carrier family 43 member 3; *slc39a4*, solute carrier family 39 member 4; *slc5a8*, solute carrier family 5 member 8; *anxa2*, annexin A2; *aqp3*, aquaporin 3; *gpr110*, G-protein coupled receptor 110; *tmprss13*, transmembrane protease serine 13; *bhmt5*, betaine-homocysteine S-methyltransferase-5; *IL8*, interlukin-8.

To validate our Illumina sequencing results, ten differentially expressed genes between LS and HS groups including four solute carrier family member genes (*slc6a15*, *slc43a3*, *slc39a4*, *slc5a8*), *annexin A2* (*anxa2*), *aquaporin 3* (*aqp3*), *G-protein coupled receptor 110* (*gpr110*), *transmembrane protease serine 13* (*tmprss13*), *betaine-homocysteine S-methyltransferase-5* (*bhmt5*) and *interlukin-8* (*IL8*) were selected for qRT-PCR analysis. The results showed that the expression trends of those genes in qRT-PCR were significantly correlated with the RNA-Seq data (R^2^ = 0.9607). On the whole, the RNA-Seq results were verified by the qRT-PCR data, indicating the reliability and accuracy of the Illumina sequencing and data analysis (Fig 1).

## Discussion

The present study is the first whole-genome scale analysis of the transcriptome of spotted sea bass, an important economical teleost which can tolerant to a broad range of salinity conditions. This RNA-Seq project not only identifies potentially differentially expressed transcripts between the two salinity environments, but also provides a large number of new annotated gene sequences in spotted sea bass, a non-model specie without any reference genome for which only a very limited number of sequences were available so far. Based on the next generation Illumina sequencing, a total of 197,550 transcripts were generated for *L*. *maculates* with 33,877 (17.15% of the total) can be matched in databases. This valuable sequence resource largely enriched the transcriptome data and prompting the genome studies of spotted sea bass.

RNA-Seq analyses have been accepted as a robust approach to assess transcriptional responses to different experimental conditions, especially in non-model organisms for which no reference genome is available [17]. For salinity adaptation study, the comparison of transcriptomic library constructed from the two salinity treatment groups enable identification of hundreds of potentially differentially expressed genes. In recent years, RNA-Seq analysis has been conducted in osmoregulatory organs (gill, intestine and kidney) for several kinds of teleost. Those studies identified several potential genes and pathways involved in salinity change [7,11, 32,33]. Although liver is not an osmoregulatory organ, it is the major source supplying energy to osmoregulatory organs for iono- and osmoregulation. An adequate and timely energy supply is a prerequisite for enzymes and transporters used in the iono- and osmoregulatory processes [20]. The energy required by the osmoregulatory organs is proved to be provided by oxidation of glucose and lactate generated from the circulation [20], and liver is the major organ involved in glucose turnover in fish [21]. Despite the fact that liver metabolism is likely enhanced during salinity adaptation, little is known about the reorganization of metabolism in liver during this process in fish. In addition, previous studies about energy metabolism in osmoregulation were focus on the physiological reaction of fish caused by osmotic pressure, as well as the energy production and expenditure (eg., oxygen consumption and ammonia excretion rates), but little was known in molecular level. In the present study, a total of 455 transcripts (with 184 up-regulated and 271 down-regulated) were identified differentially expressed in liver tissue of *L*. *maculates* between LS group and HS group. The notable number of salinity-dependent regulated genes in turn demonstrates the importance of liver in osmoregulation of spotted sea bass. We highlight several key constituents of categories and their potential functions of salinity response below.

### Energy metabolism

As we mentioned above, in order to maintain the homeostatic and osmotic balance, the iono- and osmoregulatory processes are regulated by several kinds of transporters and enzymes, and the synthesis and operation of these proteins requires a large amounts of energy [20]. Therefore, it is not surprising that we found a number of *L*. *maculates* genes with a primary role in energy metabolism were up-regulated under high salinity environment. Acetyl-CoA synthetase (*ACSS*) and cytoplasmic malate dehydrogenase (*MDH*) genes, both of which code essential enzymes utilized in various metabolic pathways, showed up-regulation in *L*. *maculates* HS group. ACSS catalyzes the ligation of acetate with CoA to produce acetyl-CoA, playing important roles in fatty acid and cholesterol synthesis and tricarboxylic acid cycle [34]. MDH is an important metabolic enzyme that reversibly catalyzes the oxidation of malate to oxaloacetate by using the NAD/NADH coenzyme system. This reaction plays an important role in the malate/aspartate shuttle across the mitochondrial membrane and in the tricarboxylic acid cycle [35]. In our study, we can infer that in order to maintain the osmotic and ionic homeostasis in the high salinity environment, the high expression levels of *ACSS* ensure that more acetyl-CoA came from energy substance metabolism can entered the Krebs cycle and the high expression of *MDH* promote the reaction continual. Besides, in our study, several genes which are likely concerned with lipid metabolism and lipid transportation were also found in DEG list. For example, Adipose triglyceride lipase (ATGL) is one of the major rate-limiting enzymes which regulate lipid storage and release in the adipocyte [36]. Previous works have discussed lipid metabolism in terms of fish osmoregulation as lipids are important energy source in fish. In fat snook (*Centropomus parallelus*), the importance of lipids for meeting the requirement of metabolism after long-term seawater (30 ppt) acclimation have been demonstrated [37, 38].

### Metabolites and ion transporters

Corresponding to the differential expressed genes engaged in energy production, a number of genes with functions for transporting molecules involved in these metabolic processes stood out as well. Along with the up-regulation of lipid metabolic enzyme mentioned above, lipid transporters including apolipoprotein B (*Apo-B*), apolipoprotein C (*Apo-C*) and apolipoprotein E (*Apo-E*) were found up-regulated in liver of spotted sea bass in high salinity group. *Apo-E* has a prominent role in lipid metabolism because of its ability to interact with lipoprotein receptors [39] and the same role for *Apo-B* which was found up-regulated in intestine of *Dicentrarchus labrax* [40]. For glucose transportation, the *GLUT* family (a specific group of the *SLC* family, *slc2a*) was identified to transport monosaccharides, polyols and other small carbon compounds across cell membranes which play a major role in glucose trafficking [20, 41]. In liver of *L*. *maculatus* in HS group, we observed up-regulation of *slc2a5/ GLUT5*, which is the key fructose transporter [42]. Another solute carrier gene, *slc5a8* (*SMCT*), belonging to the Na+/glucose co-transporter gene family, mediates a variety of mono-carboxylates including lactate, short-chain fatty acids and nicotinate [43]. The monocarboxylate transporters, *slc6a15* family that involved in amino acid transport [44] and Na/Pi co-transport system protein (*slc34a*,*nptA*) [45]which important for Pi homeostasis were also found in our DEG list, indicating they may be involved in liver osmoregulation under salinity stress.

In teleost fish, osmotic balance is maintained by coordinated water and ion transport in the intestine, gill and kidney [46]. Although the liver is not an osmoregulartory organ, several classical ion transporters were found in our DEG list, such as Na^+^-K^+^-2Cl^-^contransporter (*nkcc2*), transient receptor potential cation channel subfamily M member 5 (*trmp5*), ATP-sensitive inward rectifier potassium channel (*kcnj15* and *kcnj16*), and aquaporin 3 (*aqp3*), all of which were significantly up-regulated expressed in high salinity group (Table 4). These ion transporters were extensively studied for their roles in osmoregulatory organs [47–50]. Results showed that expression trend of those genes in *L*. *maculates* liver were in general agreement with previously reports on these gene’s roles in fishes’ osmoregulatory organs with one exception, *aqp3* expression has been found decreased in response to high salinity challenge in many teleost species [51–53], such a discrepancy may result from the different studied species and tissues.

### Signal transduction

Adaptive and acclamatory responses of fish to salinity stress depend on efficient mechanisms of osmosensing and osmotic stress signaling [54]. Instead of directly coupling osmosensors to osmotic effector proteins, large scale osmoregulatory mechanisms are operated by linking molecular osmosensors to cell signaling pathways to initiate adaptive reactions [55]. Cellular signaling pathways are activated by lig-and-receptor binding and are propagated through a number of transducer proteins via phosphorylation or dephosphorylation events [55]. In that case, osmosensory signals are not only quickly transduced within cells but also can be amplified and distributed to many types of downstream osmotic effectors [54, 55]. In our results, we found several genes involved in signals transduction were differentially expressed between LS and HS groups (Table 4), such as G-protein coupled receptor 64, cadherin-17-like, ATP P2X receptor, transmembrane protease serine 13-like and Kv channel-interacting protein 4-like which are potential osmotically regulated signaling proteins in *L*. *maculates*. They may have important functions in osmo-regulated signaling cascades which are used to amplify or deliver osmosensory signals, and then modify the functional properties of a variety of downstream osmotic effectors.

### Immune response

It is generally accepted that stresses depress the functions of the immune system in humans [56]. This connection between stresses and immune system is particularly evident in lower vertebrates [57]. In fish species, a number of immune changes have also been described after different kinds of stress treatment, and those studies suggested that the stress response will depend on the intensity of the stressor and its duration [57]. Recent work proved that if the stressor is acute and short-time, the fish immune response presented an activating phase that specially enhances innate immune responses. On the contrary, if the stressors are chronic and long-time, the immune response shows depressing effects and therefore the chances of infection may be enhanced [57]. Correspondingly, we found that several DEGs in liver transcriptome of spotted sea bass were classified into the immune system. For example, complement C1q-like protein 2, one of members in the complement system, helping destroy the pathogens and eliminate the infection, showed significantly higher expressed in LS than HS groups (Table 4). Similarity, in Asian seabass (*Latescal carifer*), complement C1q-like protein 2-like showed significant down-regulation after high salinity challenge [18]. Interleukin-8, a major pro-inflammatory cytokines which play a vital role in initiation of inflammatory responses against bacterial- and viral-infections [58], also showed differentially regulated between LS and HS groups (Table 4). Interleukin-8 was also observed appearing on the DEGs list of Asian seabass after long-time salinity challenge [18]. It may be assumed that the effects caused by chronic stressors have been mostly associated to the activity of the corticosteroid stress hormones of the hypothalamic-pituitary-interrenal axis, and in particular cortisol [57]. Further studies should be carried out to illustrate the mechanisms of immune responses linked to the salinity stress.

### Structure reorganization

Hyper or hypo-osmotic stress leads to cell shrinkage and swelling. In that case, cytoskeletal organization is notably affected by perturbations in cell volume, thus cytoskeletal proteins have been considered as putative osmosensors [59]. It is believed that cytoplasm’s osmotic strength by reorganizing the cytoskeleton structure may reflect an attempt to regain osmotic balance [60]. Correspondingly, in *L*. *maculates* liver, genes encoding multiple structural components of the cytoskeleton were also exhibit different expression patterns between LS and HS group (Table 4).

The validation results of qRT-PCR indicate the reliability and accuracy of NGS data. While both observations of NGS and qRT-PCR are limited to transcriptional level, it provides insight into future studies aimed at characterizing and comparing the contributions of salinity regulated genes in osmoregulatory and non-osmoregulatory organs to salt and water balance during salinity acclimation.

In conclusion, the research on salinity adaptation and the response to salinity stress has been long of interest not only for the important physiological significance but also for the practical application in aquaculture. Our study successfully obtained 455 differentially expressed genes in the liver of *L*. *maculatus* exposed to two salinity concentrations (5 and 30ppt). Those salinity regulated genes are associated with metabolites and Ion transporters, energy metabolism, signal transduction, immune response and structure reorganization suggesting the important role of liver for osmoregulation and salinity adaptation. The gene expression patterns and pathways provide insight into understanding the molecular mechanism of salinity acclimation and osmoregulation as well as the response to salinity stress for aquatic species.

## Supporting information

S1 Appendix Primers used for quantitative Real-Time PCR (qRT-PCR) validations. (DOCX)

S2 Appendix Annotation and functional classification of transcripts in liver of spotted sea bass. **A). Top-hit species distribution of BLASTX matches of assembled transcripts. B). Function annotation of assembled transcripts based on Gene Ontology (GO) analysis. C). Classification of assembled transcripts based on the euKaryotic Ortholog Groups (KOG) database. D)**. **Pathway assignment based on the Kyoto Encyclopedia of Genes and Genomes (KEGG) database.** Transcripts were assigned to five main categories (that include (A) cellular process, (B) environmental information processing, (C) genetic information processing, (D) metabolism and (E) organismal systems).
(TIF)

S3 Appendix Volcano plot for the differentially expressed genes shows the estimated log_2_ (fold change) (*x*-axis) against its statistical significance (*y*-axis) between LS and HS groups. Using the cutoff adjusted-p value of 0.05 (-log10 (adjusted-p value) = 1.3), a total of 455 DEGs from the upper left region (in green color, means the expressions of 271 transcripts are lower in LS group than those in HS group) and the upper right region (in red color, means the expressions of 184 transcripts are higher in LS group than those in HS group) are selected. Genes that have the adjusted-p value larger than 0.05 (in blue color) indicated there are no differentially expression between the two groups.
(TIF)

S4 Appendix Information of 455 differential expressed genes between LS and HS groups. (XLS)

S5 Appendix GO enrichment analysis of the differentially expressed genes between LS and HS groups in the liver of spotted sea bass. (TIF)
