# Supplementary material for: RNA-Seq analysis of salinity stress–responsive transcriptome in the liver of spotted sea bass (Lateolabrax maculatus)
Source: PLoS One. 2017 Mar 2;12(3):e0173238. doi: 10.1371/journal.pone.0173238 (PMC5333887; doi:10.1371/journal.pone.0173238)
Supplement: S1 Appendix — (DOCX) [file pone.0173238.s001.docx]

| Genes | Primers (5’-3’) |
| --- | --- |
| *slc6a15* | F: AAACTGCTCCGGGTATGTTG  R: GGAGAGGACACGCTTACTGC |
| *slc43a3* | F: GGATTGAACTGCGGAAAGG  R: AGACGGTTGAGCATTGGG |
| *slc39a4* | F: AACGCCTTCAATCCATGAAC  R: GCCGACCGTTTCCTACATAA |
| *anxa2* | F: AAACCCGACGACTCAACTGT  R: GCCAATGTCTGCAAAATCTACT |
| *aqp3* | F: GATCAGATAATTGGCACAGCG  R: TGGCAGGATTGACAGCATAG |
| *gpr110* | F: TCATGCTGCCTCCAGTAGAA  R: CTGCTTCCTCGTCATCAACA |
| *tmprss13* | F: CAAGTCACACGGTCAAGCAT  R: CAGCAGACCAAGCACCAGTA |
| *slc5a8(smct)* | F: CAGCGATACATCTCCTGCAA  R: AGAACATGGTGAGGCCAGAA |
| *bhmt5* | F: AAATCTCTGGGGCACAAATC  R: GCCTTCACTTCCGTCTCACT |
| *IL8* | F: TGTAGCACTCCTGGTTTTCG  R: TCCAATGGGTTTTTTCTCCT |
| *slc2a9* | F: GGAATCCAAACATACGAGACC  R: AATGAAACCGCATGACTACAA |
| *nkcc2* | F: ATCCTACGCCAAGTCTCCAG  R: GCAGCCCACCAGTTGATAA |
| *18S* | F: GGGTCCGAAGCGTTTACT  R: TCACCTCTAGCGGCACAA |
