# Supplementary figures and images for: RNA-Seq analysis of salinity stress–responsive transcriptome in the liver of spotted sea bass (Lateolabrax maculatus)
Source: PLoS One. 2017 Mar 2;12(3):e0173238. doi: 10.1371/journal.pone.0173238 (PMC5333887; doi:10.1371/journal.pone.0173238)

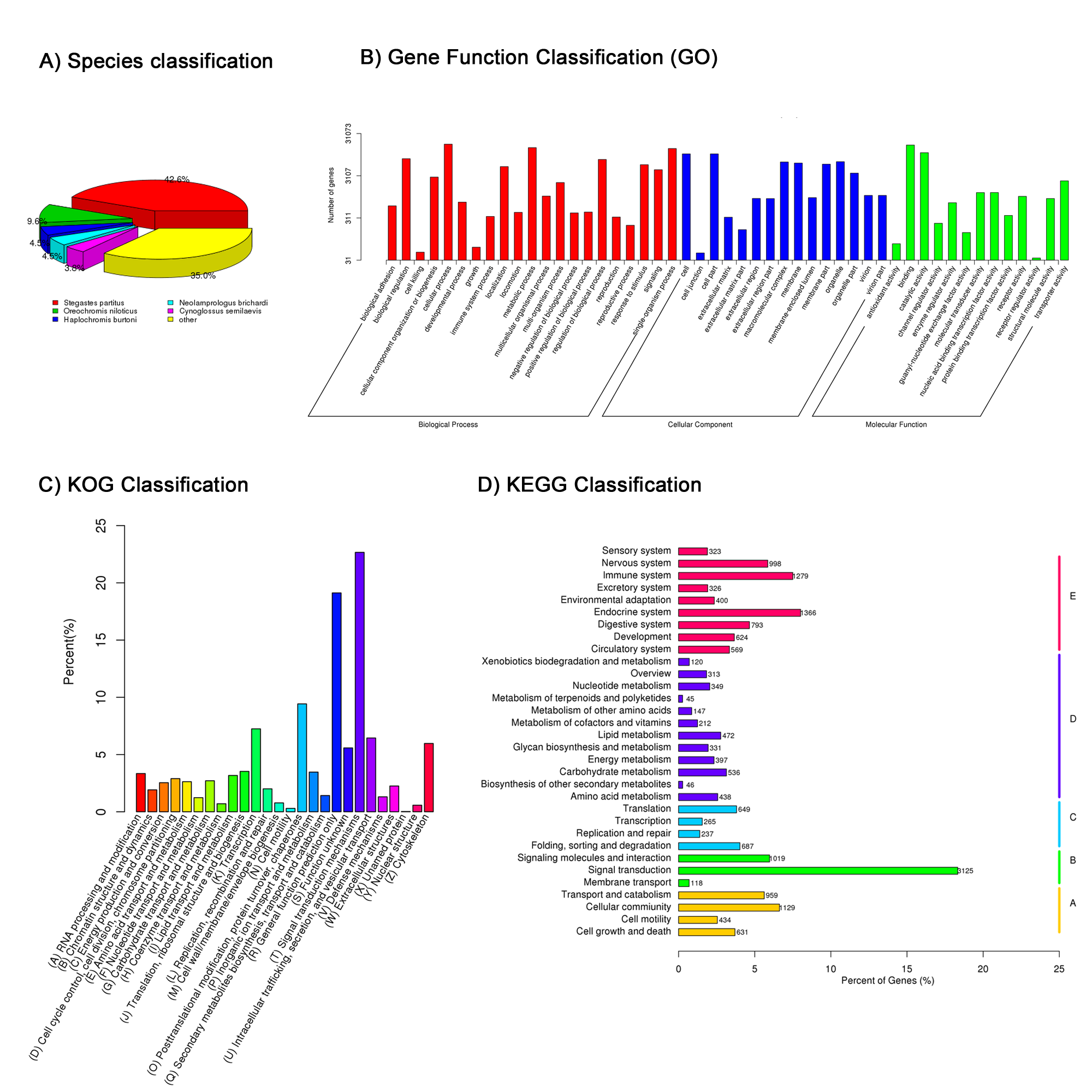

Supplement: S2 Appendix — A). Top-hit species distribution of BLASTX matches of assembled transcripts. B). Function annotation of assembled transcripts based on Gene Ontology (GO) analysis. C). Classification of assembled transcripts based on the euKaryotic Ortholog Groups (KOG) database. D). Pathway assignment based on the Kyoto Encyclopedia of Genes and Genomes (KEGG) database. Transcripts were assigned to five main categories (that include (A) cellular process, (B) environmental information processing, (C) genetic information processing, (D) metabolism and (E) organismal systems). (TIF) [file pone.0173238.s002.tif]

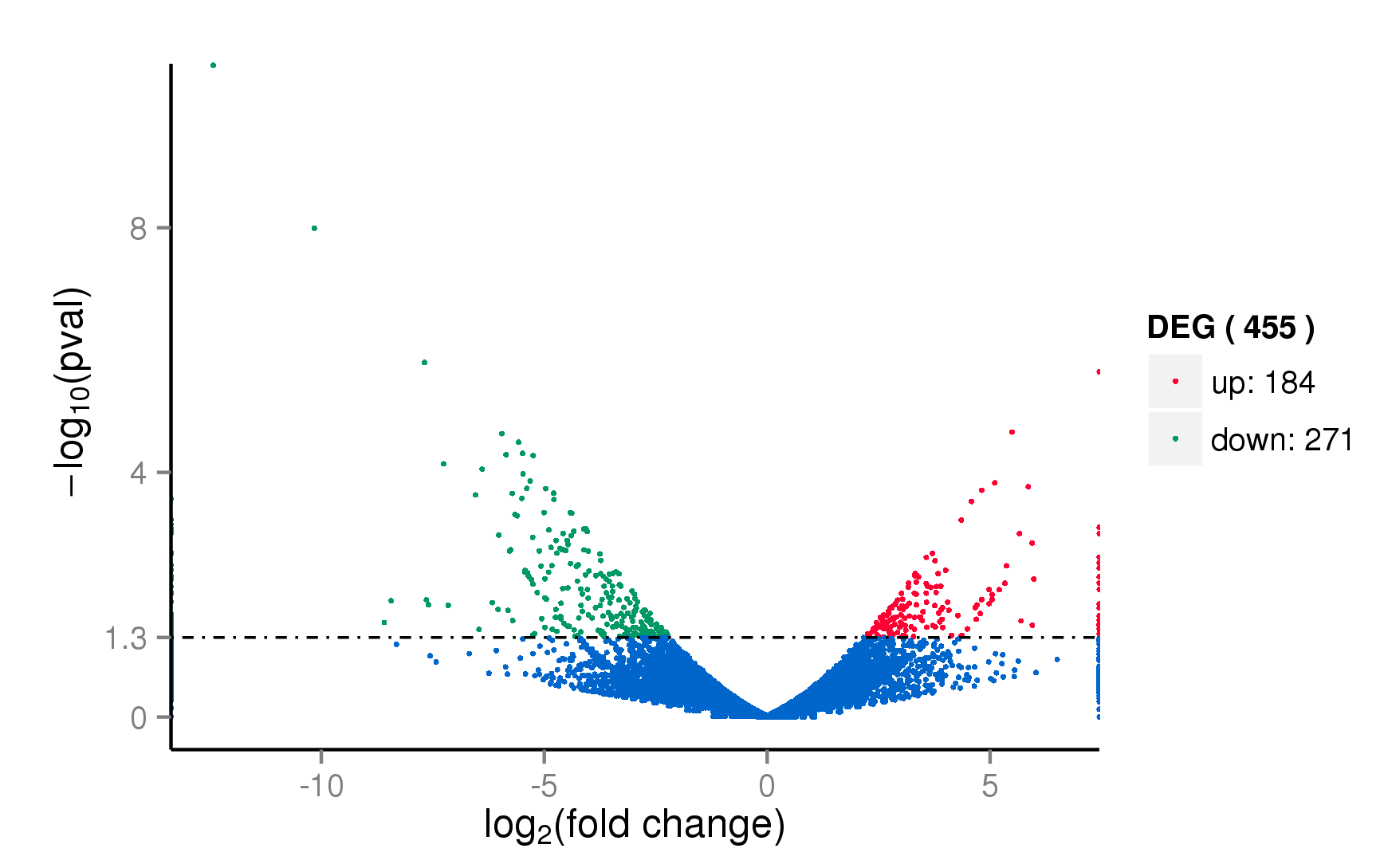

Supplement: S3 Appendix — Using the cutoff adjusted-p value of 0.05 (-log10 (adjusted-p value) = 1.3), a total of 455 DEGs from the upper left region (in green color, means the expressions of 271 transcripts are lower in LS group than those in HS group) and the upper right region (in red color, means the expressions of 184 transcripts are higher in LS group than those in HS group) are selected. Genes that have the adjusted-p value larger than 0.05 (in blue color) indicated there are no differentially expression between the two groups. (TIF) [file pone.0173238.s003.tif]

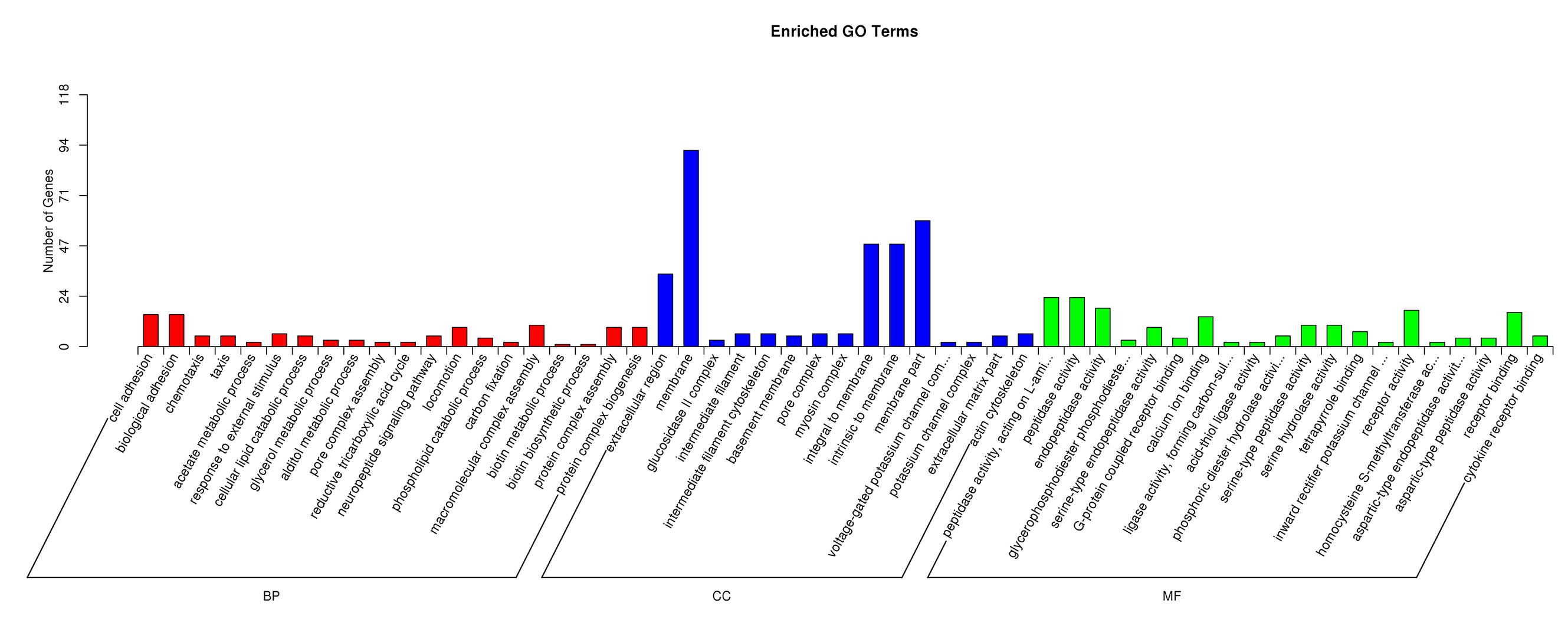

Supplement: S5 Appendix — (TIF) [file pone.0173238.s005.tif]
